# Community-based door to door census of suspected people living with epilepsy: empowering community drug distributors to improve the provision of care to rural communities in Cameroon

**DOI:** 10.1186/s12889-020-08997-8

**Published:** 2020-06-05

**Authors:** Joseph Kamgno, Jules B. Tchatchueng-Mbougua, Hugues C. Nana-Djeunga, Lynda Esso, Honorat G. Zouré, Charles D. Mackenzie, Michel Boussinesq

**Affiliations:** 1 Centre for Research on Filariasis and other Tropical Diseases (CRFilMT), P.O. Box 5797, Yaoundé, Cameroon; 2 Faculty of Medicine and Biomedical Sciences, University of Yaoundé I, Yaoundé, Cameroon; 3 Service d’épidémiologie, Centre Pasteur du Cameroun, Membre du Réseau International des Instituts Pasteur, Yaoundé, Cameroon; 4 Expanded Special Project for Elimination of NTDs (ESPEN), World Health Organization, African Regional Office, Brazzaville, Republic of Congo; 5 Filarial Programmes Support Unit (FPSU), Liverpool School of Tropical Medicine, Pembroke Place Liverpool, Liverpool, L3 5QA UK; 6 Institut de Recherche pour le Développement (IRD), UMI233/ INSERM U1175/ Université de Montpellier, 911 Avenue Agropolis, 34394 Montpellier Cedex 5, France

**Keywords:** Epilepsy, Distribution, Community drug distributors

## Abstract

**Background:**

Epilepsy is a severe neurological disorder with huge psychological, social, and economic consequences, including premature deaths and loss of productivity. Sub-Saharan Africa carries the highest burden of epilepsy. The management of epilepsy in Cameroon remains unsatisfactory due to poor identification of cases and a limited knowledge of the distribution of the disease. The objective of this study was to determine whether community drug distributors (CDDs) - volunteers selected by their communities to distribute ivermectin against onchocerciasis and who have been proven efficient to deliver other health interventions such as insecticide-treated bed nets to prevent malaria, vitamin A tablets, and albendazole to treat soil transmitted helminthiasis - can be used to reliably identify people living with epilepsy to promote better management of cases.

**Methods:**

This study was carried out in three health Districts in Cameroon. An exhaustive house to house census was carried out by trained CDDs under the supervision of local nurses. In each household, all suspected cases of epilepsy were identified. In each health district, five communities were randomly selected for a second census by trained health personnel (research team). The results of the two censuses were compared for verification purposes.

**Results:**

A total of 53,005 people was registered in the 190 communities surveyed with 794 (1.4%) individuals identified as suspected cases of epilepsy (SCE) by the CDDs. In the 15 communities where the SCE census was verified, the average ratio between the number of suspected cases of epilepsy reported in a community by the research team and that reported by the CDDs was 1.1; this ratio was < 0.8 and > 1.2 in 6 communities.

**Conclusions:**

The results of this study suggest that CDDs, who are present in about 200,000 communities in 31 Sub Saharan African countries where onchocerciasis is endemic, can be successfully used to assess epilepsy prevalence, and therefore map epilepsy in many African countries.

## Background

Epilepsy is a disorder characterized by an enduring pre-disposition to generate epileptic seizures, the latter being defined as a transient occurrence of signs and/or symptoms due to abnormal excessive or synchronous neuronal activity in the brain [1]. Epilepsy is associated with significant psychological, neurobiological, cognitive, social, and economic consequences including increased health care needs, premature deaths and loss of productivity. The number of people suffering from life-time epilepsy (i.e. with a history of epilepsy, regardless of treatment or recent seizure activity) is estimated to be 6.8 million in high-income countries and 62 million in low- and middle-income countries (45 and 17 million in rural and urban areas, respectively) [2]. The numbers of people with active epilepsy (i.e. those who may benefit from treatment) are estimated at 5.7 and 27 million in high-income and low- and middle-income countries, respectively [2]. Epilepsy represents 0.7% of the global burden of disease, as assessed by disability-adjusted life years (DALY) [3]. The median annual incidence of epilepsy - estimated to be 50.4/100,000 - is significantly higher in low- and middle-income countries (81.7/100,000) than in high-income countries (45.0/100,000) [4].

In Sub-Saharan Africa, the prevalence of epilepsy, as well as the excess mortality associated with this condition, are particularly high [5–8]. In a rural area in Cameroon, for instance, the relative risk of dying within 10 years was increased by 6.2-fold [95% CI 2.7; 14.1] in SCE [9]. In Africa, the management of epilepsy faces many difficulties, including poor availability of, and access to, antiepileptic drugs (AED) and the lack of information about disease prevalence and thus the amount of AED needed [10]. The latter issue is largely due to the paucity of trained health personnel able to accurately diagnose the condition [11, 12]. In addition, there is wide variation in the prevalence and incidence rates between and within countries, mainly due to differences in the distribution of epilepsy risk factors [5–7, 13–15]. It is therefore important to identify areas where the burden of epilepsy is particularly high to appropriately allocate personnel and financial resources and to identify local risk factors for epilepsy (particularly infectious diseases) which could be targeted for specific control measures.

In most African countries, conventional door-to-door surveys for epilepsy are often limited to selected communities. Rapid assessment strategies involving the communities themselves to assess the number of SCE could expand the epidemiological coverage of these surveys. Such strategies, including census by key informants or methods known as “participatory rural appraisal”, have been tested in several countries [16–19]. Each of these methods has advantages and limitations, even when combined in a multi-stage screening approach [20, 21]. However, they might produce rough information on regional variation in the prevalence of epilepsy and help to identify local risk factors.

From more than 20 years, a so-called “community-directed” strategy was applied to deliver drugs as part of international programmes targeting neglected tropical diseases (NTD). This approach was first tested against onchocerciasis, a parasitic disease due to *Onchocerca volvulus* which can cause blindness and severe skin manifestations. Community drug distributors (CDDs) living in the endemic communities and selected by community members are in charge of distributing ivermectin (Mectizan®) tablets once or twice a year to the whole population [22]. This decentralized, sustainable and cost-effective strategy led to a dramatic decrease in the level of infection, and probably its elimination in many foci [23]. As community-directed treatment with ivermectin (CDTI) proved to be successful, investigations were conducted to evaluate whether other community-directed interventions (CDI) could be applied [24–27]. Nowadays, CDDs are involved not only in onchocerciasis control, but also in distribution of ivermectin plus albendazole against lymphatic filariasis, of albendazole or mebendazole against soil-transmitted helminths, of praziquantel against schistosomiasis, and of vitamin A to prevent complications of deficiency [28, 29]. Involvement of CDDs in more complex interventions against malaria, tuberculosis and HIV has also been tested or considered in some countries [30–32].

In addition to the delivery of drugs, vaccines or mosquito-nets, community volunteers can help identifying subjects with visual impairment [33–37], Buruli ulcer, trachomatous trichiasis, and visceral leishmaniasis for referral for surgery or treatment [38–40], and in detecting cases of poliomyelitis [41–43] and Guinea worm [44] in conjunction with ongoing elimination programs.

The objective of the present study, conducted in 2012, was to evaluate the ability of CDDs already involved in CDTI against onchocerciasis to carry out a reliable census of SCE living in their community.

## Methods

### Study area

In Cameroon, the health system at the peripheral level is organized into Health Districts (HDs) and Health Areas (HAs). The HD is the operational unit for the implementation of public health programs. It is comprised of HAs, defined by the presence of an integrated health center covering an average of 10 communities. The study was carried out in the Ndikiniméki and Monatélé HDs (Centre region), and in Pouma HD (Littoral region). The Monatélé and Pouma HDs are limited to the north by the Sanaga River, and the Ndikiniméki HD is limited to the east by the Mbam River, the main tributary of the Sanaga. These three HDs are highly endemic for onchocerciasis [45, 46] and CDTI has been in place since 1999. An association between onchocerciasis and epilepsy was demonstrated in the neighboring Bafia HD [47]. Much of the treatment and care for epilepsy in the study HDs is supported by caregivers associated with religious organizations. All communities in all HAs in the selected HDs were included in the study.

### Study design

The study was conducted from 2011 to 2012 in areas of Cameroon with historically high prevalences of epilepsy [48–50]. Given the CDDs’ experience visiting households for many years, we assumed that they had established a bond of trust with the population to whom they provide various type of community cares and would therefore be able to gather reliable data, even on a stigmatized disease such as epilepsy that people are usually hiding [8]. To assess accuracy, the data collected by the CDDs were compared with those obtained subsequently by the team of the Centre for Research on Filariasis and other Tropical Diseases (CRFilMT), so-called research team. During the verification by the research team, all surveyed communities were geo-localized (geographical coordinates were collected at the center of each community) using the Global Position System (GPS) in order to map the distribution of epilepsy in the study HDs.

### Training of CDDs and health personnel

The process started with the sensitization of the administrative, health, and traditional authorities, as well as the general population, about the procedures and objectives of the study. All CDDs of each health district attended the training session organized by the District Medical Officer and the nurse head of the Health Center. The CDDs, the nurses head of Health Centers, and a member of the HD office were trained for 2 days by the research team on the objectives and the procedures of the study. Participants in the training received basic information on the etiology and various types of epilepsy, the ways to prevent stigmatization of SCE, and how to conduct a census of the general population, record the SCE and complete the registration forms. Sessions were also organized in the Health Centers to provide practical experience to surveyors.

The training relied on the questionnaire developed by Preux while carrying out studies on epilepsy in tropical countries [50] (see questions 7–8 in Additional file 1). Based on this questionnaire, simulation and explanation of different type of crises were done, in order to capacitate them to explain to the populations and to be able to collect the types of crisis in communities. The different type of crises explained were: (i) the falls with loss of consciousness, (ii) shaking or uncontrollable movements of one or more limbs, (iii) loss of contact with the entourage, and (iv) strange body sensations, visual illusions, auditory or olfactory (odors) [51].

### Census procedures for the CDDs

In each study community, an exhaustive house to house survey was carried out over a period of 1 week by the trained CDDs. Each CDD recorded information from 20 households, representing approximately 100 inhabitants. The process was supervised by the nurse head of the Health Center for the community, who did not formally participate in the census. In each household, the head of the household (HoH) (or his/her spouse/partner or any other adult family member who was able to provide accurate answers if the latter was absent during the survey) was asked to provide the name, age and occupation of each household member, including those who were temporally absent during the visit of the survey teams (either CDDs or research team). This information was recorded on a specific census form (Additional file 1).

Next, the HoH was asked whether any household member suffered or is suffering from epilepsy. In the case of a positive answer, the standardized questions to define the type of crises [50] were asked to elicit demographic data, as well as the circumstances and presentation of the epileptic crisis (Additional file 1). According to the criteria of the International League Against Epilepsy at the time of the study, suspected cases of epilepsy (SCE) were defined by a history of two or more unprovoked seizures occurring at least 24 h apart [52]. Isolated seizures associated with fever were excluded.

### Verification by the trained health personnel (research team)

In each of the three HDs, five communities were randomly selected by drawing of lot for verification of the number of SCEs, without any assumption on a potential difference between communities. For time constraint reasons, only one of these communities was used to confirm the census data for the total population in each HD. The verification team consisted of trained health personnel (medical doctors and nurses specialized in public health), and the census procedures used for verification, both for the general population and the SCEs, were identical to those used by the CDDs, though independent.

### Data analysis

The prevalence of epilepsy with 95% confidence intervals estimated from the data collected by CDDs in each community were calculated at both the HD and HA levels. A concordance analysis was performed to compare the performance of CDDs and that of the trained health personnel in enumerating the general populations and SCE [53]. Intrinsic (sensitivity, specificity) and extrinsic (positive and negative predictive values) parameters were used to (i) compare the census of the general population in each community between CDDs and research team, and (ii) assess whether the proportions of SCE by CDDs were similar to those by the research team, the results obtained by the trained personnel being used as the reference. Also, Kappa statistics was used to evaluate the concordance of census between CDDs and research team in three communities where both teams conducted census of general population and SCE [53]. All statistical analyses were performed using STATA 12.1 software (STATA Corporation, college Station, TX, USA). Since epilepsy is usually associated with onchocerciasis, CDDs-based point prevalence of epilepsy was illustrated according to altitude and distance to hydrographic network (both indicative of black fly breeding sites) using the software ArcGIS, version 10.2, ESRI Inc.

## Results

### Prevalence of epilepsy measured by the CDDs

**Fig. 1:**
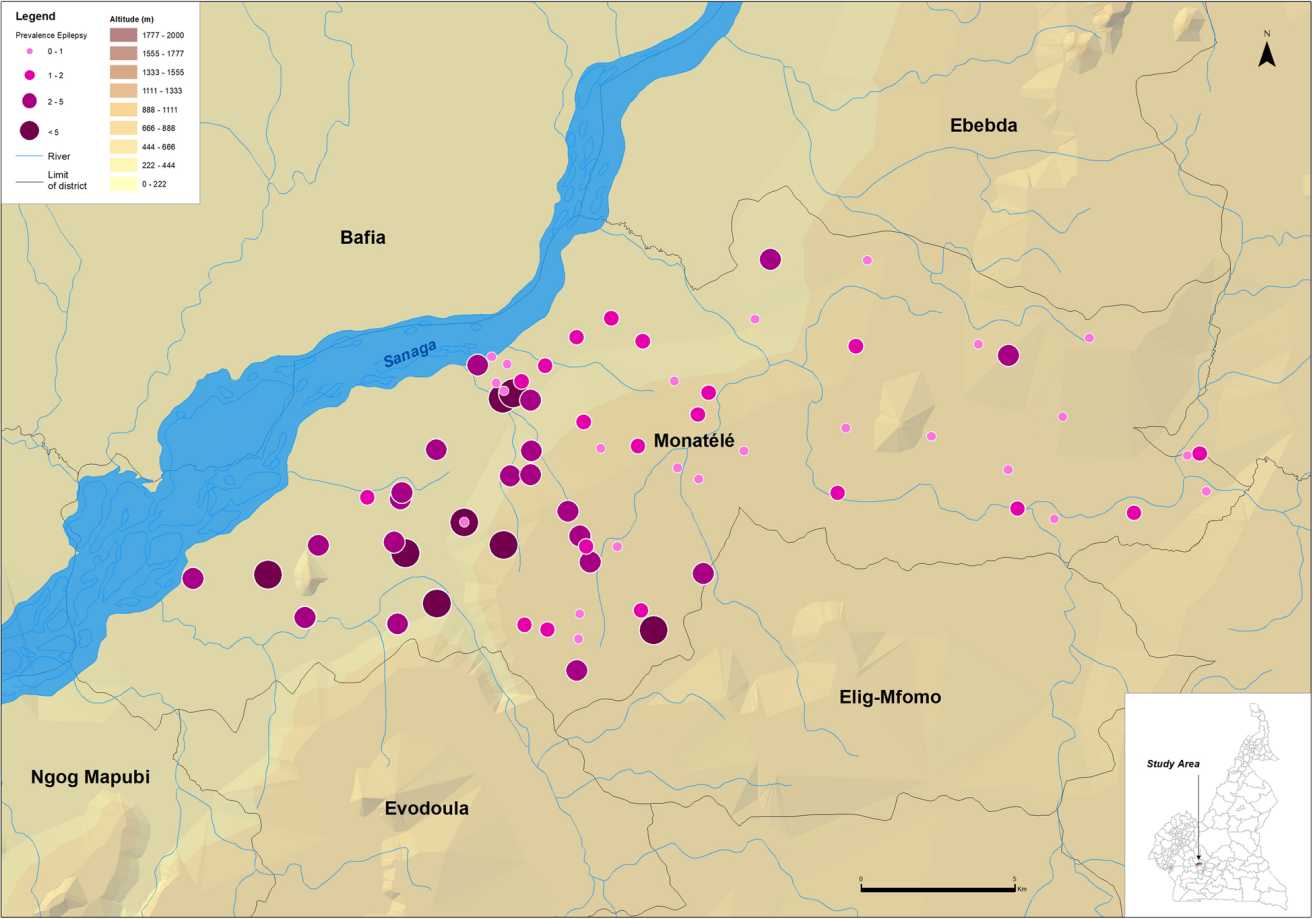
Prevalence of CDDs-based suspected cases of epilepsy in the Monatélé Health District (Centre Region, Cameroon). Each dot represents a point prevalence of epilepsy at community level and is represented according to altitude and distance to hydrographic network both indicative of black fly (vector of *Onchocerca volvulus*) breeding sites

**Fig. 2:**
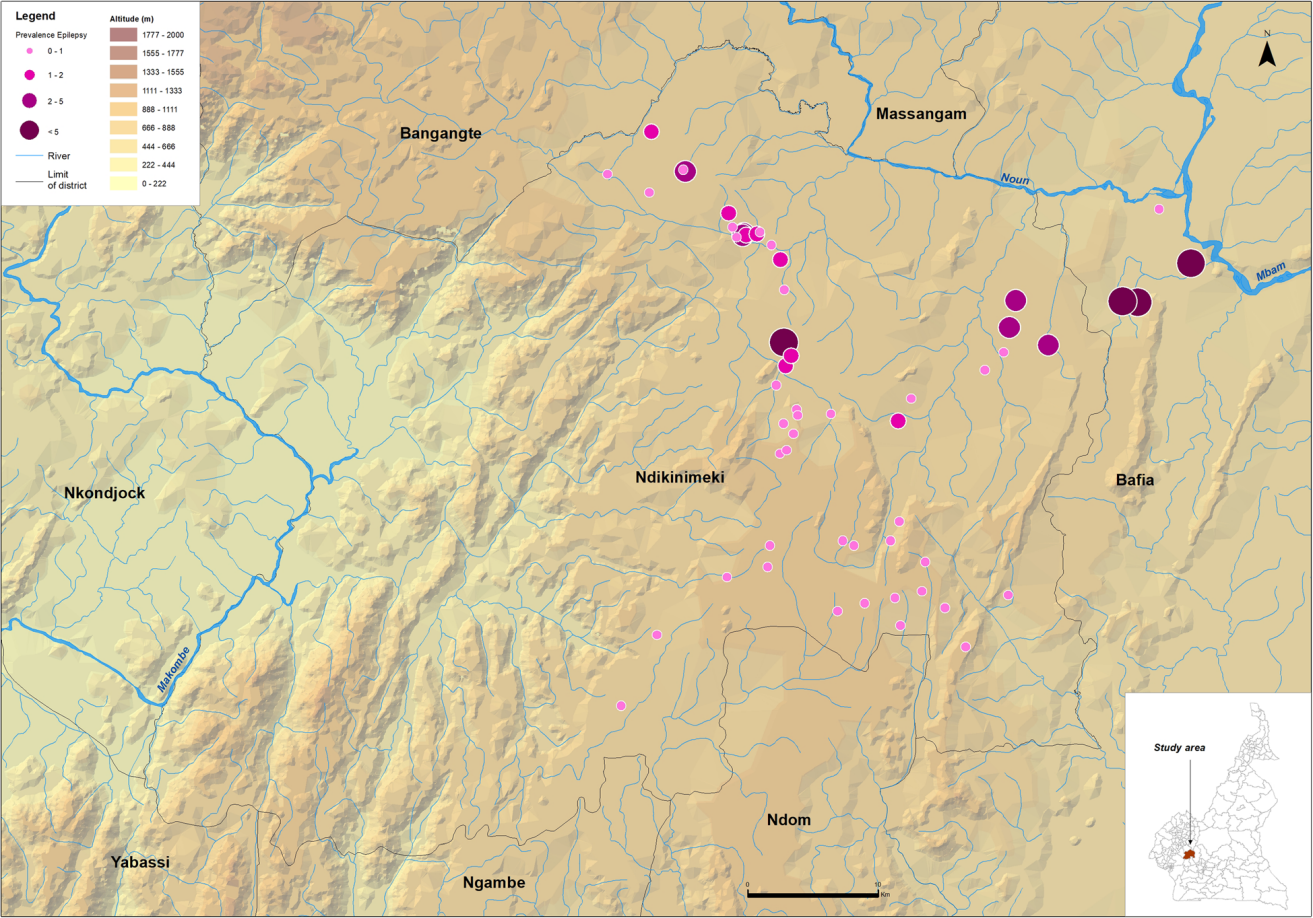
Prevalence of CDDs-based suspected cases of epilepsy in the Ndikinimeki Health District (Centre Region, Cameroon). Each dot represents a point prevalence of epilepsy at community level and is represented according to altitude and distance to hydrographic network both indicative of black fly (vector of *Onchocerca volvulus*) breeding sites

**Fig. 3:**
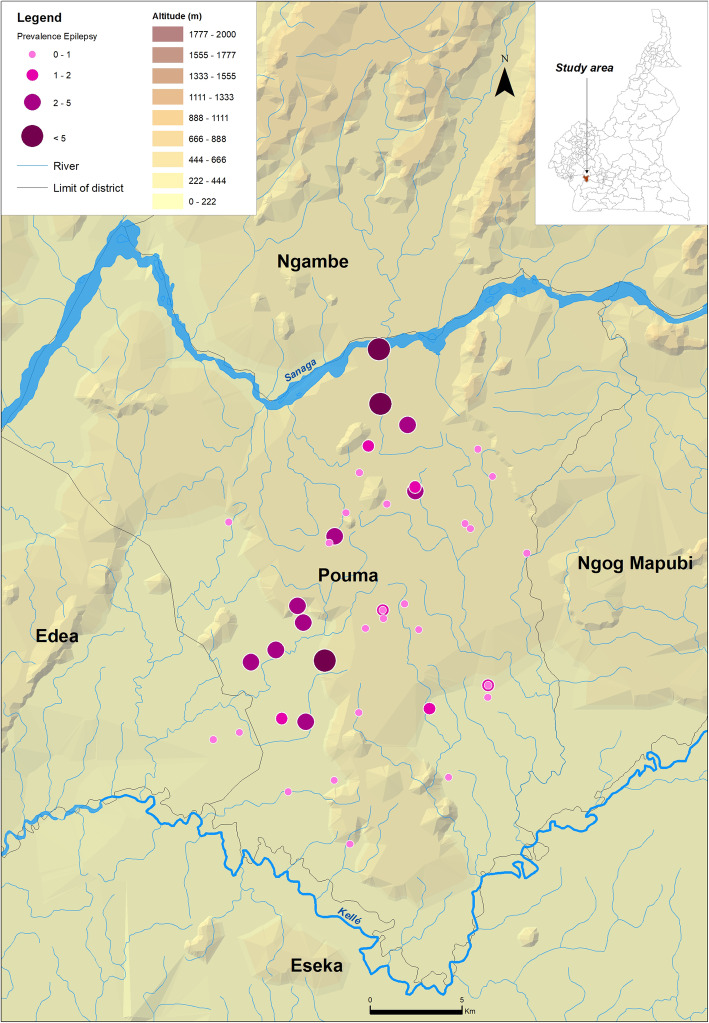
Prevalence of CDDs-based suspected cases of epilepsy in the Pouma Health District (Littoral Region, Cameroon). Each dot represents a point prevalence of epilepsy at community level and is represented according to altitude and distance to hydrographic network both indicative of black fly (vector of *Onchocerca volvulus*) breeding sites

The census was conducted in 185 communities located in 19 HAs (8 in Monatélé HD, 6 in Ndikiniméki HD and 5 in Pouma HD) (Table 1) by a total of 490 CDDs among which 62.0% were males. The median number of people covered by a CDD was 97.3, with an interquartile range (IQR) equal to 93.0. In 1–2 weeks (depending on the population size), the CDDs registered a total of 53,044 people in the targeted communities and recorded 766 SCEs (1.4%). The prevalence of SCEs was very variable within the HDs and even within specific HAs. The mean prevalence of SCEs was higher than 2.0% in three HAs of the Monatélé HD (Eyeng-Meyong, Monatélé and Nlong-Bong) and two HAs of the Pouma HD (Nkonga and Song Simouth) (Table 1). Figures 1, 2 and 3 show the location of each surveyed community, its distance from the hydrographic network (especially the *Simulium* breeding sites) and the prevalence of SCEs.

**Table 1:** Number of villages surveyed in each health area, and total population and suspected cases of epilepsy (SCE) recorded by the CDDs in these villages

Health district	Health area	No. villages surveyed	No. people recorded	No. SCE	Percentage of SCE per community		
					Mean	Min	Max
Monatélé	Eyeng-Meyong	4	821	22	2.7	0	5.2
	Monatélé	23	10,214	238	2.3	0	8.1
	Mvomekak II	10	4321	40	0.9	0	1.7
	Ngomo	6	2593	19	0.7	0.3	1.6
	Nkog-Bong	6	3556	26	0.7	0	1.8
	Nkolkosse	9	3439	31	0.9	0	3.3
	Nlong-Bong	12	3368	109	3.2	1.9	6.0
	Tala	8	2506	49	2.0	0.9	4.8
	All	78	30,818	534	1.7	0	8.1
Ndikiniméki	Boutourou	6	2093	39	1.9	0	4.6
	Makenene	9	3721	36	1.0	0	2.5
	Ndikiniméki	22	5241	41	0.8	0	7.5
	Ndokowanen	3	951	0	0	0	0
	Nitoukou	13	1376	1	0.1	0	0.6
	Nyokon	6	1859	24	1.3	0	2.8
	All	59	15,241	141	0.9	0	7.5
Pouma	Makob Log-Bako	10	1528	20	1.3	0	6.1
	Nkonga	7	1184	28	2.4	0	8.9
	Pouma-Centre	10	1646	11	0.7	0	4.7
	Saint-André	12	1555	7	0.5	0	1.9
	Song Simouth	9	1072	25	2.3	0	6.6
	All	48	6985	91	1.3	0	8.9
All		185	53,044	766	1.4	0	8.9

At the community level, 7 of the 78 communities surveyed in the Monatélé HD had a prevalence of SCE exceeding 5.0%, with the highest values in Levem (8.1%), Mong (6.9%), Nkol-Melong (6.5%) and Nlongbong II (6.0%) (see data per community in Additional file 2). In the same HD, the prevalences ranged from 3.0 to 5.0% in 15 other communities. In the Ndikiniméki HD, only one of the 59 communities surveyed had a prevalence of SCE exceeding 5.0% (Nomena: 7.6%) and 3 others had prevalences between 3.0 and 5.0% (Kiboum I: 4.7%; and Kiboum II: 4.4% and Ndikoti: 4.8%). In Pouma HD (48 communities surveyed), the prevalence of SCE exceeded 5.0% in 4 communities (Logbassom: 8.9%, Sakbayeme III: 6.6%; Ngompem-Nkanla: 6.1% and Sakbayeme II: 5.6%) and ranged between 3.0 and 5.0% in one community (Pouma Centre administratif: 4.8%).

Different types of crises were reported by CDDs, including falls with loss of consciousness (79.8%), shaking or uncontrollable movements of one or more limbs (62.7%), loss of contact with the entourage (67.2%), and strange body sensations, visual illusions, auditory or olfactory (40.7%).

### Validation survey by trained health personnel

The validation survey by the research team aimed at verifying the census of the general population as well as that of the SCE. This validation was conducted approximately 3 months after the census by CDDs, and was organized in a total of 15 communities (among which the census of the general population was verified in three of them), with a total population of 5895 inhabitants, corresponding to 11.1% of the total population of the three health districts.

In the three communities (one in each health district) selected for verification of the general population census data, the results obtained by CDDs were in general lower than those obtained by the trained health personnel (864 vs 1040 people, respectively), including 342 vs. 595 people in Kiboum I (Ndikiniméki HD), and 230 vs. 252 people in Nkondjock II (Pouma HD). The difference was particularly marked in Kiboum I, where 42.5% of the population was missed by the CDDs.

In contrast, in the 15 communities (five in each health district) where the number of SCEs was verified by the research team, the difference between the number of cases recorded by the CDDs and that recorded by the health personnel exceeded two in only one community, Nkolkosse (columns CDD+ and Team+ in Table 2). The kappa coefficient (0.91) show almost perfect agreement between census performed by CDDs and research team. The number of SCE recorded by the health personnel was higher than that recorded by the CDDs in 8 communities, the same in 2 communities, and lower in 5 communities (Table 2). The average ratio between the number of SCE reported in a community by the research team and that reported by the CDDs was 1.1 (range 0.5 to 2.0, with a ratio < 0.8 and > 1.2 in only 6 communities). At HD level, the ratio was higher (and thus more SCEs were missed by the CDDs) in Monatélé HD (1.4) than in Ndikiniméki and Pouma HDs (1.1 in both HD). The sensitivity of diagnosis made by the CDDs (using the health personnel as the gold standard) was 48.3%, ranging from 42.5% in Nidikiniméki HD to 60.0% in Pouma HD, with a positive predictive value of only 55.1%, ranging from 48.6 to 68% depending on the HD. The most frequent type of crisis reported by the validation team were tonic-clonic crises (79.8%), SCEs exhibiting partial crises and with absence of crises were also reported in 10.4 and 9.8% of cases, respectively.

**Table 2:** Suspected cases of epilepsy (and prevalence of epilepsy) recorded by the CDDs (CDD+) and by the research team (Team+) in the villages where the verification was conducted, resulting prevalences, and ratio between the prevalences

District	Health area	Village	Pop*	No. SCEs		Prevalence		Ratio	CDD+ ^§^ Team+	CDD + ^μ^ Team-	CDD-^β^ Team+
				CDD+	Team+	CDD	Team				
Monatélé	Eyen-Meyong	Nkol-Feb**	292	3	4	1.03	1.37	1.3	2	1	2
	Mvomekak	Ebolmongo II	626	11	10	1.76	1.60	0.9	8	3	2
	Ngomo	Ebanga	513	3	5	0.58	0.97	1.7	3	0	2
	Nkog-Bong	Nkog-Bong	896	3	5	0.33	0.56	1.7	3	0	2
	Nkolkosse	Nkolkosse	512	5	10	0.98	1.95	2.0	1	4	9
	All		2839	25	34	0.88	1.20	1.4	17	8	17
Ndikiniméki	Boutourou	Boneck I	449	10	12	2.23	2.67	1.2	3	7	9
	Boutourou	Kiboum I**	342	15	17	4.39	4.97	1.1	9	6	8
	Makenéné	Mock Sud	459	6	7	1.31	1.53	1.2	3	3	4
	Ndikiniméki	Ndikitole	363	4	2	1.10	0.55	0.5	2	2	0
	Nyokon	Kinding Ndjabi	339	0	2	0	0.59	NA	0	0	2
	All		1952	35	40	1.79	2.05	1.1	17	18	23
Pouma	Makob Lob Bako	Ngompem-Nkanla	114	7	6	6.14	5.26	0.9	4	3	2
	Nkonga	Nkonga A	152	1	0	0.66	0	0	0	1	0
	Pouma Centre	Song Woga	325	2	2	0.62	0.62	1.0	2	0	0
	Saint André	Nkondjock II**	230	3	3	1.30	1.30	1.0	3	0	0
	Song Simouth	Sakbayeme II	283	5	4	1.77	1.41	0.8	0	5	4
	All		1104	18	15	1.63	1.36	0.8	9	9	6
ALL			5895	78	89	1.32	1.51	1.1	43	35	46

* Population recorded by the CDDs

** Villages where both the population census and the census of SCE were verified by the project team

§ SCE recorded by both the CDDs and the research team (CDD+ Team+)

μ SCE recorded by the CDDs only (CDD+ Team-)

β SCE recorded by the research team only (CDD- Team+)

## Discussion

Epilepsy is a very common condition in Africa, where some of the highest prevalence and burden in the world have been recorded. This is likely due to an important cocktail of potential causes of this condition in Africa, such as (i) viral, bacterial and parasitic infections with potential neurological involvement [6, 54–59], as well as (ii) cranial trauma consecutive to pregnancy and/or childbirth complications, and road accidents [60, 61].

Compounding the high prevalence and burden of epilepsy in Africa is the large treatment gap, defined as the proportion of people with epilepsy who require treatment but do not receive it. Indeed, a systematic review showed that this gap exceeds 75% in low-income countries, and that it is greater than 95% in 7 of the 11 African countries included in the study [62]. The main causes of this treatment gap have been related to the health systems in Africa, which are plagued by inadequate skilled manpower, high costs of treatment and the unavailability of drugs [63]. In this context, it is important to identify areas where the prevalence of epilepsy is particularly high, so that efforts can be focused where patients’ needs are the greatest and local preventable causes of epilepsy may be identified.

Published data on the distribution of epilepsy in Cameroon is very scanty and limited to a few communities. A major factor limiting the availability of data is the paucity of personnel to conduct epidemiological assessments. To address this problem, this study aimed to evaluate the capacity of CDDs to conduct the census of SCE at the same time as the general census of population. Discussions conducted during the training confirmed the results of other studies [64–67], demonstrating that most people in the communities are well aware of the disease and suggesting that CDDs could easily conduct the census of SCEs.

The present study demonstrated that the sensitivity of CDD assessment to identify SCEs was relatively low. However, since the number of individuals falsely suspected to be epileptic and the number of SCE who were missed by the CDDs were also low, the prevalences of epilepsy calculated from data collected by the CDDs were not statistically different from those obtained by the research team. However, it is worth to mention that the number of SCEs recorded by both CDDs and the research team was quite low, and the power of the comparison test likely low. Despite this limitation, it is clear that CDDs can provide, at relatively low cost, a picture of the burden of epilepsy at the level of communities, HAs or HDs, and reveal areas where the prevalences are particularly high. Indeed, CDDs or other community health workers could be used to identify SCEs at first line, and the latter referred to health care professionals for a clinical diagnosis of epilepsy for SCE confirmation and furthermore appropriate treatments. This approach has been recently suggested for a comprehensive management of epilepsy in onchocerciasis-endemic areas, and was based on a decentralized epilepsy care, with simplified approaches for the diagnosis and treatment of epilepsy by non-physicians, under the supervision of physicians or specialists [68]. It is also important to note that the population census (measurement of the denominator) has to be carefully checked because in some instances the population size can be under- or overestimated by the CDDs. The census of the general population and of SCE by CDDs is however perfectible and can be refined through additional trainings and exercises. Indeed, census of population is conducted at a yearly basis by CDDs in the framework of onchocerciasis control or other community public health interventions, given them the opportunity to sharpen their census of SCE and adjust their denominators.

The prevalences of SCEs identified during this study were very high in some of the HAs surveyed, particularly in Monatélé and Ndikiminéki HDs. These results are in accordance with previous results in the region [52]. Considering the sociologic and demographic impacts of epilepsy in the neighboring Bafia HD [8], special interventions are clearly required in the study area. Given the previously demonstrated relationship between onchocerciasis and epilepsy [48], CDTI should certainly be reinforced and, over time, may reduce the incidence of epilepsy of this region. Since the effect of ivermectin on pre-existing epilepsy is less evident, better access to anti-epileptic drugs (AEDs) should also be prioritized. However, the prevalence of epilepsy reported in this study is in general low as compared to those reported in the review by Bugembe and colleagues [65]. It is worth to mention that these data were collected after about 15 years of ivermectin-based mass treatments against onchocerciasis. There are mounting evidences of a strong relationship between onchocerciasis and epilepsy, and a temporal relationship between these two diseases has been recently demonstrated [66]. This likely means that if the prevalence and intensity of onchocerciasis infection decreased as a consequence of mass treatments, the incidence of epilepsy might follow the same trends. In addition, it was recently demonstrated that the SCE prevalence decreased after multiple rounds of ivermectin-based MDA [69, 70], the reduction of *Onchocerca volvulus* transmission recently reported leading to a decrease in the number of new cases of epilepsy, and consequently to an age shift among SCE observed [71].

### Limitation

As stated above, slight discrepancies were observed between the census of the general population as well as that of SCE reported by CDDs and research team. These discrepancies can be explained, at least partly, by the fact that (i) people interviewed by the CDDs and the research team in each household were not always the same. The weight of these errors in census either of general population or SCE cannot be estimated since the information was not collected; (ii) verification censuses were conducted approximately 3 months after the censuses by CDDs, and slight variation in the populations, both general and that of SCEs, consecutive to births, deaths or migrations might be expected.

It is likely that these discrepancies can be larger if one wants to scale up this strategy. As such, data reported by the CDDs are not 100% reliable, some SCE needing treatment might be missed, and some individuals not suffering from epilepsy wrongly enrolled. To overcome this situation, a validation of SCE identified by CDDs should be done by trained health personnel before initiating treatments. Once initiated, the “ripple effect” of these treatments might be useful to identify missing cases who might have been hiding in communities.

## Conclusions

This study revealed that community health workers or CDDs may be able to perform the census of SCE, especially in rural areas, though some substantial differences were sometimes found while validating their work and the power of comparison test was low. More research in other settings should therefore be conducted to confirm this observation. As demonstrated for other health interventions, training and monitoring are key to the collection of reliable data. Should this strategy be used at a larger scale, identification of areas where specific etiologies of epilepsy are present could help guide policy makers in prioritizing areas of greatest need. Finally, the use of community personnel as a repository for AEDs at the community level supervised by health personnel should be explored since this approach might help increasing epilepsy treatment coverage and compliance.

## Supplementary information

**Additional file 1.** Census form. Form used by the CDDs to perform census of suspected cases of epilepsy in the three selected health districts.

**Additional file 2.** Dataset. Community-based aggregated data of census of suspected cases of epilepsy in each health district.

## Data Availability

The data collected during this study are presented in the manuscript and in the supplementary documents (Supplementary material). Raw data collected are available from the corresponding author at the Centre for Research on Filariasis and other Tropical Diseases (CRFilMT).

## Abbreviations

CDDs: Community Drug Distributors
HDs: Health Districts
AEDs: anti-epileptic drugs
SCE: Suspected cases of epilepsy
HoH: head of household
SCE: people living with epilepsy
