# Supplementary material for: Community-based door to door census of suspected people living with epilepsy: empowering community drug distributors to improve the provision of care to rural communities in Cameroon
Source: BMC Public Health. 2020 Jun 5;20:871. doi: 10.1186/s12889-020-08997-8 (PMC7275343; doi:10.1186/s12889-020-08997-8)
Supplement: Supplementary file 1 — Additional file 1. Census form. Form used by the CDDs to perform census of suspected cases of epilepsy in the three selected health districts. [file 12889_2020_8997_MOESM1_ESM.zip › Additional file 1_English versionR3.docx]

**GENERAL INFORMATION SHEET**

1. Health District : ………………………………………………………………
2. Village : ………………………………………………………………………………….
3. House N° : ……………………………………………………………………………
4. Respondent (Head of household = 1 ; other = 2) : |………….|
5. Head of household’s name : ………………………………………………………………………………………………
6. Number of household members : |………….|

| **N°** | **Name and Surname** | **Age** | **Sex** | **Activity/Profession** |
| --- | --- | --- | --- | --- |
|  |  |  |  |  |
|  |  |  |  |  |
|  |  |  |  |  |
|  |  |  |  |  |
|  |  |  |  |  |
|  |  |  |  |  |
|  |  |  |  |  |
|  |  |  |  |  |
|  |  |  |  |  |
|  |  |  |  |  |

1. Are there any known epileptic person in the family?
   1. |………….| (Yes = 1 ; No = 2)
   2. If yes, how many ? |………….|
2. Are there people in the family who repetitively have :
3. Falls with loss of consciousness? (Yes = 1 ; No = 2) |………….|
4. Loss of contact with the entourage? (Yes = 1 ; No = 2) |………….|
5. Uncontrollable shaking or movement of one or more members? (Yes = 1 ; No = 2) |………….|
6. Strange bodily sensations, visual auditory or olfactory (odors) illusions? (Yes=1 ; No=2) |….….|
7. How many ? |………….|
