# Supplementary material for: Community-based door to door census of suspected people living with epilepsy: empowering community drug distributors to improve the provision of care to rural communities in Cameroon
Source: BMC Public Health. 2020 Jun 5;20:871. doi: 10.1186/s12889-020-08997-8 (PMC7275343; doi:10.1186/s12889-020-08997-8)
Supplement: Supplementary file 1 — Additional file 1. Census form. Form used by the CDDs to perform census of suspected cases of epilepsy in the three selected health districts. [file 12889_2020_8997_MOESM1_ESM.zip › Additional file 1R3.docx]

**FICHE DE RECENSEMENT GENERAL DE LA POPULATION**

1. District de santé de : ………………………………………………………………
2. Village : ………………………………………………………………………………….
3. Maison N° : ……………………………………………………………………………
4. Personne interrogée (Chef de famille = 1 ; autre = 2) : |………….|
5. Nom du Chef de Famille : ………………………………………………………………………………………………
6. Nombre d’habitants dans la maison : |………….|

| **N°** | **Noms et prénoms** | **Age** | **Sexe** | **Activité/Profession** |
| --- | --- | --- | --- | --- |
|  |  |  |  |  |
|  |  |  |  |  |
|  |  |  |  |  |
|  |  |  |  |  |
|  |  |  |  |  |
|  |  |  |  |  |
|  |  |  |  |  |
|  |  |  |  |  |
|  |  |  |  |  |
|  |  |  |  |  |

1. Existe-t-il des personnes connues épileptiques dans la famille ?
   1. |………….| (Oui = 1 ; Non = 2)
   2. Si Oui, combien ? |………….|
2. Existe-t-il dans la famille des personnes qui présentent de manière répétitive :
3. des chutes avec pertes de connaissance ? (Oui = 1 ; Non = 2) |………….|
4. des pertes de contact avec l’entourage ? (Oui = 1 ; Non = 2) |………….|
5. des secousses ou mouvements incontrôlables d’un ou des membres ? (Oui = 1 ; Non = 2) |………….|
6. des sensations corporelles étranges, illusions visuelle, auditive ou olfactive (odeurs) ? (Oui=1 ; Non=2) |….….|
7. Combien ? |………….|
